# Research on an innovative design and evaluation method of Chinese tea sets based on GT-AHP-FCE

**DOI:** 10.1371/journal.pone.0302005

**Published:** 2024-04-11

**Authors:** YanXiao Zhao, Basyarah Hamat, Tao Wang, SongEn Wang, Leah Ling Li Pang

**Affiliations:** 1 Razak Faculty of Technology And Informatics, Universiti Teknologi Malaysia, Kuala Lumpur, Malaysia; 2 Anyang Institute of Technology, Anyang, Henan, China; Nuclear Science and Technology Research Institute, IRAN, ISLAMIC REPUBLIC OF

## Abstract

**Aims:**

In order to explore new consumer demands for Chinese tea set products, propose an innovative tea set product design and evaluation method to improve the user experience and satisfaction of the produced tea sets, thereby promoting the development of the tea set market and the promotion of tea culture.

**Methods:**

Firstly, grounded theory (GT) was used to analyze interview data to extract consumer demand indicators and construct a design evaluation hierarchical model. Secondly, the Analytical Hierarchy Process (AHP) was used to calculate the weights of the indicators, determine their priority of importance, and obtain several indicators that have a greater impact on the tea set design to guide innovative design practice. Lastly, the tea set design schemes were evaluated using the fuzzy comprehensive evaluation method to select the optimal design scheme and also to act as a guideline for further design optimization.

**Conclusion:**

This study explores the innovative design and evaluation method for tea set products based on GT-AHP-FCE and validates the feasibility of this approach through a practical example of tea set design inspired by “The Classic of Mountains and Seas.”. It provides innovative theoretical and practical guidance for designers of subsequent tea set products and also provides a new path for the inheritance and innovation of traditional culture.

## Introduction

Chinese tea culture has a long history. Tea sets, as the utensils used in the preparation and serving of tea, are the fundamental component of tea culture and have significant market value [1]. With the improvement of production technology and diversified lifestyle changes in the recent years, consumers’ diversified demand for tea sets has also increased vastly [2]. However, there is a gap between the existing Chinese tea set designs and consumer demands. Problems such as the homogenization of tea set products and the lack of Chinese cultural elements and characteristics have weakened the product’s appeal to consumers and market competitiveness [3]. This is due to the lack of systematic, effective, and feasible design methods that could guide designers, followed by designers merely imitating and applying the designs from other product ideas. In addition, various academic researches on tea set design have been conducted prior to this study. For example, Zhang Z Q [4], Jiang S S [5], and Min G P [6] studied tea set design from the perspectives of Maslow’s hierarchy of needs theory, emotional design, and experiential design and recommended that tea set design should provide users with a better emotional experience. Zheng D [7] conducted an in-depth analysis of the application of advanced and basic modeling technology in the design of ceramic tea sets, and used digital models to discover problems with existing ceramic tea sets and optimize them. Zhang et al. [8] discussed the innovative design principles of smart car tea sets based on user experience and applied these principles into the product design. The above-mentioned existing literature provides ideas for the innovative development of tea set design, but lacks in-depth research on consumer needs. Additionally, there is a gap in the research on design decisions and comprehensive evaluation methods of tea set products. Thus, this paper will explore the design decisions and comprehensive evaluation methods of tea set products based on user demands. In the user analysis stage, most existing research conduct interviews, questionnaire surveys, and other methods to obtain user demand indicators. Then, the categorization of indicators by the researchers themselves to determine evaluation indicators. This process has a certain degree of subjectivity and lacks the guidelines of qualitative research theory. Therefore, this study introduces grounded theory in the process of establishing evaluation indicators to scientifically and systematically analyze the data obtained from the semi-structured interviews. The analyzed data is then used to establish a comprehensive evaluation hierarchy model of tea set products. Grounded theory is a more scientific qualitative research method that searches for the core concepts that reflect the nature of things on the basis of systematically collecting data, which then constructs relevant substantive theories through the connections between these concepts. The advantage of applying grounded theory lies in the fact that it can systematically analyze, refine, and summarize the raw data to find the essential issues of the research object. Grounded theory has gradually been used by many scholars in research in various fields. For example, Chao C. et al. [9] built an emotional design model for future intelligent products based on grounded theory. Lin L. et al. [10] used grounded theory to analyze the factors that affect farmers’ willingness to sell agricultural products through new media and combined the analysis results to provide suggestions for solving practical problems in agricultural product sales. Seesawang J. et al. [11] used grounded theory to develop a theory about the personal risk perception experience of elderly hypertensive male patients in rural Thailand. Grounded theory combined with the semi-structured interview method has a certain degree of freedom and openness and is able to explore on indicators that are overlooked in current theories. It is more scientific and objective than the inductive summarization method, which relies on the researcher’s experience. In the comprehensive evaluation process, many existing studies use the analytical hierarchy process (AHP) and the fuzzy comprehensive evaluation (FCE) methods individually or a combination of both for design evaluations. Due to its theoretical completeness, structural rigor, and simplicity in problem-solving, the analytic hierarchy process has shown significant advantages [12], especially in solving multi-objective and multi-criteria decision-making problems [13]. As a result, it has been widely applied in various fields. For example, Guo S. et al. [14] studied the application of the mathematical idea of the fuzzy comprehensive evaluation method in community museums. Guo S. established a community museum quality evaluation system, and constructed a fuzzy comprehensive evaluation model of community museum experience. Fan [15] used a method combining the analytic hierarchy process and fuzzy comprehensive evaluation to evaluate the hazards associated with multiple subsystem failures in a complex maglev bogie system. Wang et al. [16] used the analytic hierarchy process to construct a hierarchy model for the design of cultural and creative lamps for the World Cup to guide the lamp design practice. Then, Wang et al. used the fuzzy comprehensive evaluation method to evaluate the World Cup cultural lamp design solutions to select the best design solution. Based on the above, it is shown that the analytic hierarchy process can be used to quantify user needs and prioritize demand indicators, which helps to clarify the design direction of the product. The fuzzy comprehensive evaluation method can be used for program evaluations and optimizations in selecting the optimal design program and to further design and improve the program. The Analytic Hierarchy Process provides a scientific, flexible, and straightforward analytical decision-making method for evaluating complex problems with multiple objectives and multiple criteria [17, 18]. It has the advantages of combining qualitative and quantitative aspects and being hierarchical and systematic [19]. In addition, this method also ensures a more scientific and precise analysis of the multi-level demand indicators. In product conceptual design, the utilization of the AHP method enables the hierarchical decomposition of qualitative design decision factors, and weights of indicators are obtained through quantitative calculations to systematically rank the indicators one by one [20]. In summary, AHP can provide decision-makers with comprehensive evaluation criteria, weights, and analyses, thereby reducing the risk of decision bias. The application of AHP can offer a clearer, more concrete, and more feasible guidance for product conceptual design decisions. Therefore, the analytic hierarchy process was employed in this study to quantify and rank the demand indicators which will provide effective guidance for subsequent tea set designs. The fuzzy comprehensive evaluation method was utilized to comprehensively assess the design schemes and select the optimal one. The novel comprehensive evaluation method for tea sets integrating GT-AHP-FCE proposed in this study aims to improve the objectivity and rationality of the design and evaluation process. This method will serve as a theoretical reference for subsequent designers, assisting in design decision-making and solution evaluation optimization. Hence, facilitating the production of modern tea set products that better meet consumer demands.

## Materials and methods

### Theoretical overview

#### Grounded theory

Grounded Theory (GT) which was developed by Glaser and Strauss in 1967, is a qualitative research method that rises from phenomena to theory [21]. Its primary mode is to effectively organize and synthesize the original data obtained from interviews and give rise to a system theory, thus abstracting core concepts that can convey the essence of a phenomena [22]. The main operating procedures of grounded theory are: discover and select problems; analyze and code the collected data step by step; establish theories and test the saturation of the theories; and finally, obtain effective conclusions corresponding to the problems [23]. In addition, open, axial, and selective coding of data are the key steps in developing a theory [24, 25].

#### Analytic hierarchy process

The Analytical Hierarchy Process (AHP) is an analytical decision-making method proposed by American scholar Professor Saaty that can systematize and hierarchize complex multi-objective problems [26, 27]. It quantifies qualitative indicators through pairwise comparison to calculate the weightage values and total ranking of the indicators at each hierarchy [28, 29], thus achieving design decisions [30].

#### Fuzzy comprehensive evaluation method

The fuzzy comprehensive evaluation method proposed by Professor Zadeh in 1965 uses fuzzy mathematical principles to conduct an overall evaluation and analysis of things or evaluation objects [31]. The fuzzy comprehensive evaluation method transforms qualitative evaluation into quantitative evaluation and can systematically solve problems that are difficult to quantify or have a certain degree of ambiguity [32, 33]. In the design field, the fuzzy comprehensive evaluation method is often used to score and evaluate solutions to find further optimization directions for the solution or to compare multiple solutions to select the best solution [34].

### Tea set design evaluation process framework

This research focuses on the discussion of product design evaluation methods and does not involve any relevant experimental research on clinical, animal, human tissues nor biological samples. In this research, humans only participated in the interview survey, and all participants were adults who participated voluntarily. This study recruited human participants to participate in interviews based on the principle of voluntary participation. Before the study began, all participants were informed in advance of the purpose of the study, the interview process, the use of the interview data, and the rights of the participants. The written consent was also obtained from the participants. All the participants gave their consent to participate in this study by signing the informed consent form (see S1 Appendix). This study does not involve discussions about individuals’ religious beliefs, racial identities, political views, sexual orientations, financial information, or any other personal privacy topics. All data and information are collected and recorded anonymously and do not involve any violations of human participants’ privacy, dignity, health, nor human rights. Thus, all research methods and procedures in this article comply with ethical principles and regulatory requirements and have been approved for exemption from ethical review.

**Fig 1:**
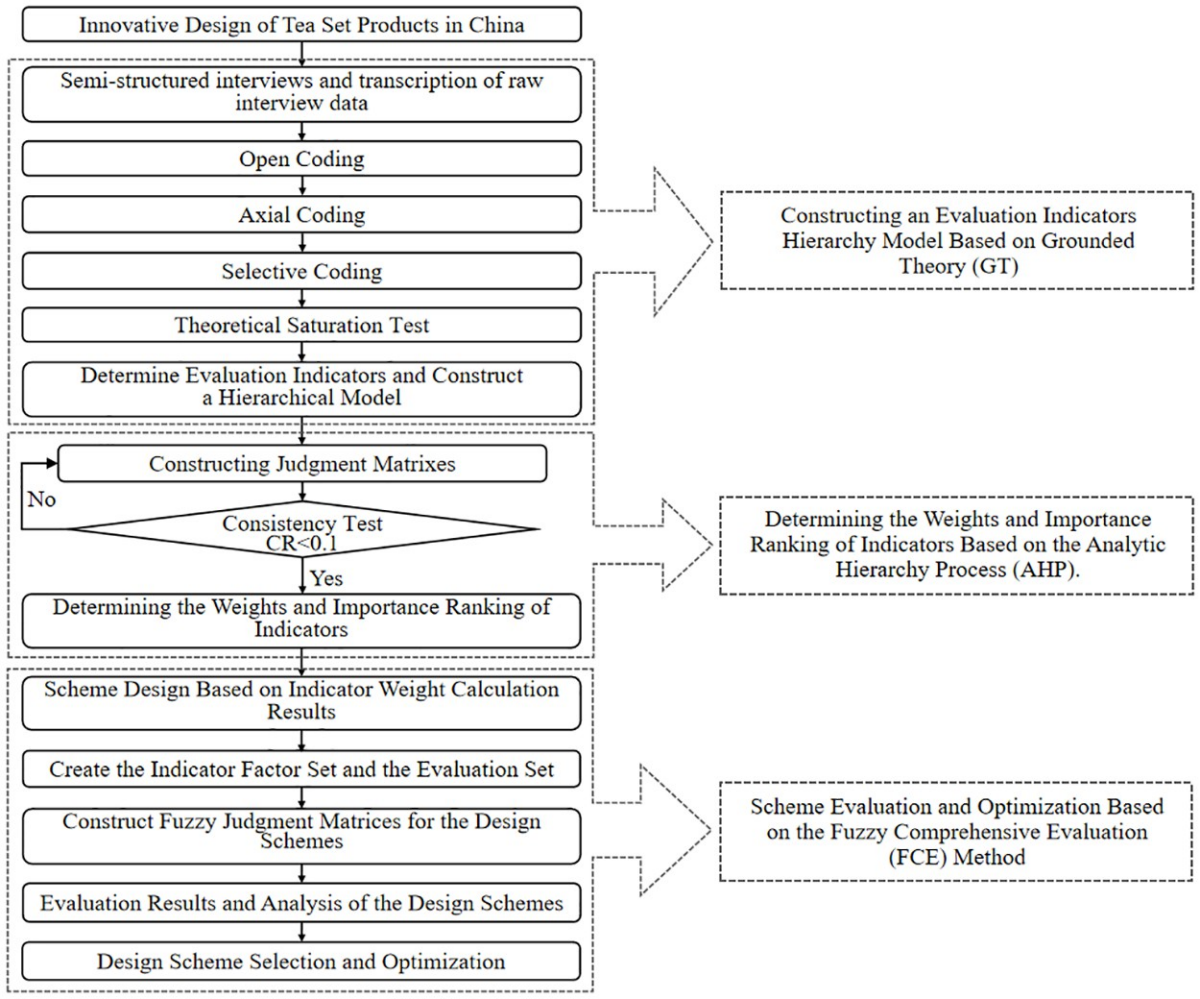
The design and comprehensive evaluation process of tea sets.

The overall process of the novel tea set comprehensive evaluation method proposed in this article can be divided into four phases: obtaining consumer demand indicators and constructing a hierarchical model; quantifying and prioritizing the indicators; preliminary product scheme design based on indicator ranking; and comprehensive evaluation and selection of the schemes. In the first phase, semi-structured interviews were conducted to gather consumers’ current and potential demands for modern tea set products. Next, the interview data was analyzed using grounded theory and gradually coded to extract user demand indicators to construct a hierarchical model. The second phase involves constructing judgment matrices based on the Analytic Hierarchy Process (AHP) and determining the weightage values and priority rankings of the indicators at each level. In the third phase, tea set schemes were designed based on key demand indicators with higher weightage values. In the fourth phase, the fuzzy comprehensive evaluation method was employed to comprehensively evaluate the design schemes, select the optimal scheme, and further clarify the optimization guidelines for the selected scheme. The design and comprehensive evaluation process of tea sets is shown in Fig 1.

## Results

### Determine evaluation indicators and the hierarchy model

#### Selection of the respondent sample

To assure the scientific, comprehensive, and representative nature of the original data collection, this study adopted a diversified expert sampling strategy to ensure the acquisition of comprehensive and multi-angle professional knowledge, opinions, and experiences. This study includes semi-structured interviews with 10 participants, comprising of tea set product designers with extensive experience, professors in the field of Chinese tea culture research, consumers with prior tea set product purchasing experience, and sales staff with many years of experience in selling tea set products.

The demographic information of the participants is described in detail as follows: (1) Two designers with over 10 years of experience in tea set product design. These designers possess extensive industrial knowledge and expertise in tea set product design, along with unique insight and understanding of consumer needs and preferences. They are able to offer valuable professional perspectives to help this study better understand and obtain consumer demand indicators for Chinese tea set products. (2) One professor in the field of Chinese tea culture research who also serves as a tea set product designer. He has outstanding research achievements in the field of tea culture. He possesses in-depth knowledge of the history, traditions, and rituals of Chinese tea culture, coupled with experience in tea ware product design. This expert can provide comprehensive support for this study by offering professional insights into the tea culture background, consumer demands for tea set products, aesthetic concepts, and more. (3) Five consumers who have purchased tea set products more than three times. They consist of different genders, age groups, and educational backgrounds, and have rich consumption experiences and diverse opinions (composition of consumer participants: different genders: 3 male, 2 female; different age groups: 1 person aged 18–25, 1 person aged 26–35, 2 people aged 36–50, 1 person aged over 50; different education qualifications: 1 person with high school education or below, 2 people with undergraduate degrees, 1 person with a graduate degree, 1 person with a doctoral degree). These consumers have directly interacted with tea set products and may have formed preferences, demands, and feedback about the products. They can provide direct opinions that reflect consumers’ demands for tea set product design. (4) Two sales staff with more than 10 years of experience in tea set product sales. Sales staffs often engage with numerous consumers and possess a certain understanding of consumer preferences and needs. They are able to share their experiences related to common consumer questions, feedback, as well as purchasing motivations encountered during their sales process. In addition, they are able to share their own observations and insights into tea set product market trends.

Interviewing the aforementioned diverse array of professionals can assist in generating rich discussions, gathering pertinent professional knowledge and experience from multiple perspectives, and collecting diverse opinions. Such interviews aid in gaining a more comprehensive and in-depth insight into consumer demands, thus ensuring the validity of the research results.

#### Primary data collection

The interviews conducted in this study targeted tea set product designers (a professor in the field of Chinese tea culture research who concurrently serves as a tea set product designer is regarded as a designer), consumers who have purchased tea set products more than three times, and sales staff. Although the purpose of the interviews is the same, the questioning approach for each group will differ due to the diverse identities of the interviewees, resulting in different interview questions as well. Therefore, an interview guideline is developed for each respondent group(refer to Tables 1 to 3) before the start of the interview process to ensure that the collected data is detailed and comprehensive. Subsequently, interviews were conducted with the participants based on the prepared interview guidelines. Respondents were encouraged to provide answers freely during the interviews without any inducements. Each interview lasted approximately 25 to 30 minutes per interviewee. After obtaining consent from the interviewees, the interviews were recorded using an audio recording equipment.

**Table 1:** Interview guideline with tea set consumers.

Serial No.	Open-ended Topic Questions	Probing Questions
1	Do you often use tea set products in your life?	How often do you use tea set products?
2	What are the reasons that usually make you choose to buy a tea set product?	Could you share in detail your previous experience in purchasing tea set products?
3	Based on your experience purchasing and using tea sets, what are your thoughts on the operating experience of tea set products?	
4	Do you have any demands on the appearance, material, and color of tea set products?	
5	What are your thoughts on the cultural features of Chinese tea sets?	What types of traditional cultural content interest you more?
6	What other problems do you think tea set products have that trouble you, or what aspects need to be optimized?	

**Table 2:** Interview guideline with tea set product designers.

Serial No.	Open-ended Topic Questions	Probing Questions
1	Approximately how many times have you participated in the design of tea set products in your work?	Can you share how you learned about consumers in your previous design work?
2	Based on your understanding of consumers, what do you think consumers’ demands are for the functional operability of tea set products?	
3	Based on your understanding of consumers, what do you think consumers’ demands are for the appearance, material, and color of tea set products?	
4	Based on your understanding of consumers, do you think consumers have demands for the cultural features of Chinese tea sets?	What specific demands do consumers have for the cultural features of Chinese tea sets?
5	Based on your experience in designing tea sets and your understanding of consumers, what other problems do you think tea set products have or what aspects need to be optimized?	

**Table 3:** Interview guideline with tea set product sales staff.

Serial No.	Open-ended Topic Questions	Probing Questions
1	In your sales work, do you often receive comments and experience feedback from consumers about tea set products?	Based on the feedback you received, what factors in existing tea sets will affect consumer satisfaction?
2	What do you think consumers’ demands are for the functional operability of tea set products based on your contact and understanding with consumers?	
3	What do you think consumers’ demands are for the appearance, material, and color of tea set products?	
4	Do you think consumers have demands for the cultural features of Chinese tea sets?	What specific demands do you think consumers have for the cultural features of Chinese tea sets?
5	What other problems do you think tea set products have or what aspects need to be optimized based on your contact and understanding with consumers?	

#### Open coding

After conducting the interviews, the audio recordings were transcribed and analyzed by coding the data using the Nvivo 12 software. To minimize potential subjectivity in the research process and ensure the objectivity and reliability in the coding of interview content, this study adopted a multi-person cross-coding approach whereby multiple researchers independently code the same data materials. This process is followed by continuous reviews, comparison of coding processes and outcomes among different coders to identify potential discrepancies, and collective discussions and negotiations to ensure consistency and accuracy of the coding process. Additionally, prior to commencing coding with Nvivo, this study established clear coding standards and guidelines to guide the coding process. Training sessions were conducted for the coding team members to ensure consensus on coding standards and procedures, thus promoting systematic and standardized coding practices.

Open coding is the process of decomposing, conceptualizing, and categorizing the data word by word in an objective manner while avoiding the influence of personal factors [35]. This study derived 11 categories of tea set product design demands through continuous analysis and summarization of the original interview data. The process of open coding is shown in Table 4.

**Table 4:** Open coding process.

Representative statements of original information	Initial Concept	Category
The handles of some tea sets are not easy to grasp or require a large-angle tilt when pouring water, which is laborious.	The structure and position of the teapot handle should be convenient for use.	Easy-to-operate teapot handle
Some tea sets are prone to tipping over and accidentally burning my hands when handling them.	The structure of tea sets should have good stability, which makes them difficult to fall over.	Structural stability
The elegant and simple color will be more in line with the quiet and peaceful atmosphere of drinking tea and will also look more comfortable.	The color coordination is elegant and rustic, creating a comfortable atmosphere.	Elegant and comfortable color matching
Sometimes, when drinking tea, I can drink some tea leaf residue, which affects the taste.	Tea sets need to be more effective at filtering tea residues.	Fully filter the tea residue
Many existing Chinese tea sets do not reflect the characteristics of its long-standing traditional culture and lack differentiation from products from other countries.	Should reflect the characteristics of China’s long-standing traditional culture.	Traditional cultural characteristics
We prefer that the tea set material is smooth and textured and does not look cold and stiff.	The material has a good texture and is smooth and comfortable.	Smooth and affinity material texture
The water column coming out of the spout of the teapot is thin, and sometimes the spout is half-blocked, resulting in water splashing.	Prevent blocking or splashing of water from the spout.	Discharges water smoothly and steadily
In many existing Chinese tea sets, we rarely see symbols or patterns that reflect traditional culture, and it is difficult to see what cultural connotations they contain.	Elements and symbols that reflect cultural connotation should be incorporated into the tea set design.	Use of cultural symbols
The appearances of many tea sets look old-fashioned, not unique and interesting, making them not good-looking.	The appearance needs to be innovative, fashionable, and interesting.	Novel and beautiful appearance
After updating the tea set at home, it would be a pity to throw away the old tea set that I replaced, but it does not have any collectible or cultural commemorative value that would justify me keeping it.	By incorporating cultural contents that have storytelling to enhance the collection and commemorative value of tea sets.	Culturally monumental
Some materials will change color and fade after being used for a long time. I am afraid that it will be harmful to my health.	The tea set material should be durable and non-toxic.	Material safety and durability

#### Axial coding

Axial coding is used to determine the main categories of the research problem by further analyzing the logical correlations between various concepts and sub-categories in the data. By further summarizing and refining the 11 sub-categories obtained in the process of open coding into itemized content, four main categories were identified: functionality, safety, aesthetics, and cultural. The main process is shown in Table 5.

**Table 5:** Axial coding process.

Main category	Sub-categories	Connotation
Functionality	Discharges water smoothly and steadily	The teapot spout discharges water smoothly and steadily, preventing blockages or splashes.
	Easy-to-operate teapot handle	Teapot handles are easy to grip and pour tea
	Fully filter the tea residue	Filters fine tea residues more effectively
Safety	Material safety and durability	Safe, non-toxic, and durable tea set material
	Structural stability	Reasonable structural design makes it not easy to fall over
Aesthetics	Elegant and comfortable color matching	The colors are not overly bright and obtrusive and look comfortable.
	Smooth and affinity material texture	The material is affinity, comfortable, and has a good texture.
	Novel and beautiful appearance	The appearances are distinctive, interesting, and aesthetically appealing.
Cultural	Use of cultural symbols	Use symbols and patterns that reflect cultural connotations
	Traditional cultural characteristics	Reflect China’s unique traditional cultural content to enhance product differentiation.
	Culturally monumental	Enhance the collection and commemorative value of tea sets by endowing them with cultural associations.

#### Selective coding

Selective coding is used to analyze the logical relationships of the categories obtained in axial coding and explore their typical path relationships and connotations. The process is shown in Table 6.

**Table 6:** Selective coding process.

Typical path relationships	Nature of relationship	Connotation
Functionality→User satisfaction→Design solutions	Intermediary relationship	Whether functionality is good affects user satisfaction and thus affects design decisions.
Safety→User satisfaction→Design solutions	Intermediary relationship	Whether safety is reliable affects user satisfaction and thus affects design decisions.
Aesthetics→User satisfaction→Design solutions	Intermediary relationship	Aesthetics affects user satisfaction and thus affects design decisions.
Cultural→User satisfaction→Design solutions	Intermediary relationship	Cultural affects user satisfaction and thus affects design decisions.

#### Theoretical saturation test

The coding process was conducted again according to the above three-level coding steps by using the three copies of original data reserved in advance. The results did not yield new concepts and categories, proving that the theory has reached the saturation point.

#### Construction of the tea sets evaluation indicators hierarchical model

**Fig 2:**
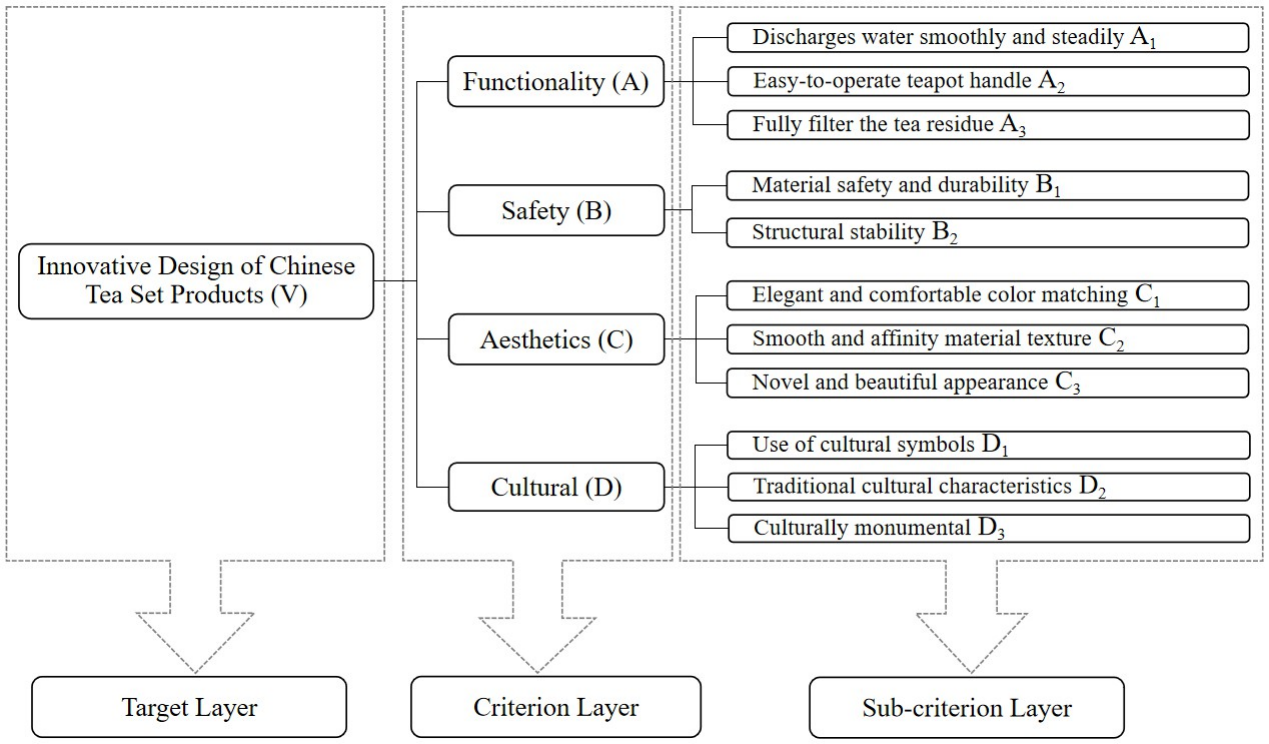
Chinese tea set product evaluation indicators hierarchical model.

Through further review and comparison of relevant previous research literature [36, 37], this study discovered commonalities between the indicators obtained and coded through interviews and some of the indicators mentioned in the literature pertaining to tea set product design [38]. Moreover, the indicators acquired in this study are more comprehensive, thus complementing and enriching the content of previous studies in the literature. Lastly, this study integrated the interview data with relevant literature, removed duplicate and inapplicable information, and finally constructed a hierarchical model of tea set products using 4 main categories and 11 sub-categories as design evaluation indicators. The evaluation hierarchical model of tea set products is shown in Fig 2.

### Determine the weight and ranking of evaluation indicators

#### Constructing a judgment matrix

Constructing the judgment matrix refers to a two-by-two comparison of the relative importance of indicators at the same level in the hierarchical model [39]. In order to quantify the judgment matrix, the scaling method from 1 to 9 was used to assign weights to the indicators [40] (scales and meanings are shown in Table 7). The numerical values were used to express the different degrees of importance between multiple indicator elements. The indicators at each level were scored and evaluated by the respondent samples selected above. This was followed by multiple rounds of discussions and summarizations to finally obtain all the fuzzy judgment matrices and the weight values of each indicator, as shown in Tables 8 to 13.

**Table 7:** Judgment matrix index importance level numerical scale table.

Scale value	Importance Level	Implication
1	Equally important	Indicator *i* is of equal importance compared to indicator *j*
3	Slightly important	Indicator *i* is marginally important compared to Indicator *j*
5	Significantly important	Indicator *i* is significantly more important than Indicator *j*
7	Extremely important	Indicator *i* is extremely important compared to Indicator *j*
9	Absolutely important	Indicator *i* is absolutely important compared to Indicator *j*
2,4,6,8	Eclectic value	The importance level is between two adjacent levels
1/2,1/3…1/9	Inverse comparison	If the importance scale of indicator *i* over indicator *j* is “n”, the inverse comparison is “1/n”.

**Table 8:** Judgment matrix and weight values for the target level.

V	A	B	C	D	Weights(w)
A	1	1/3	2	1/2	0.16509
B	3	1	3	2	0.44454
C	1/2	1/3	1	1/3	0.10721
D	2	1/2	3	1	0.28317

**Table 9:** Judgment matrix and weights for functional criteria.

A	*A*_1_	*A*_2_	*A*_3_	Weights(w)
*A*_1_	1	1/3	1/2	0.16378
*A*_2_	3	1	2	0.53896
*A*_3_	2	1/2	1	0.29726

**Table 10:** Judgment matrix and weights for safety criteria.

B	*B*_1_	*B*_2_	Weights(w)
*B*_1_	1	2	0.66667
*B*_2_	1/2	1	0.33333

**Table 11:** Judgment matrix and weights for functional criteria.

C	*C*_1_	*C*_2_	*C*_3_	Weights(w)
*C*_1_	1	2	1/4	0.20141
*C*_2_	1/2	1	1/5	0.11795
*C*_3_	4	5	1	0.68064

**Table 12:** Judgment matrix and weights for functional criteria.

D	*D*_1_	*D*_2_	*D*_3_	Weights(w)
*D*_1_	1	1/3	1/4	0.12262
*D*_2_	3	1	1/2	0.32024
*D*_3_	4	2	1	0.55714

**Table 13:** Axial coding process.

Criterion layer	Weights	Sub-criterion layer	Weights	Combined weights	Ranking
A	0.16509	*A*_1_	0.16378	0.02704	9
		*A*_2_	0.53896	0.08898	5
		*A*_3_	0.29726	0.04906	7
B	0.44454	*B*_1_	0.66667	0.29636	1
		*B*_2_	0.33333	0.14818	3
C	0.10721	*C*_1_	0.20141	0.02159	10
		*C*_2_	0.11759	0.01256	11
		*C*_3_	0.68064	0.07297	6
D	0.28317	*D*_1_	0.12262	0.03472	8
		*D*_2_	0.32024	0.09068	4
		*D*_3_	0.55714	0.15777	2

#### Consistency test

In order to ensure that there is no self-contradiction in the evaluator’s thinking during the process of constructing the judgment matrix, a consistency test is required after solving the judgment matrix and the weights of the evaluation indicators. The test steps are as follows:

1. Use formula 1 to calculate the maximum eigenvalue:
  $$\begin{array}{c} \lambda_{\text{max}}=\frac{1}{n}\sum_{i=1}^{n}\frac{{\left(A\omega \right)}_{i}}{\omega_{i}} \end{array}$$ (1)
2. Use formula 2 to calculate the consistency index:
  $$\begin{array}{c} CI=\frac{\lambda_{\text{max}}-n}{n-1} \end{array}$$ (2)
3. Use formula 3 to calculate the consistency ratio:
  $$\begin{array}{c} CR=\frac{CI}{RI} \end{array}$$ (3)

In the formula, RI is the random consistency index, and the RI values of different order matrices are shown in Table 14.

**Table 14:** RI values of matrix order 1–9.

1	2	3	4	5	6	7	8	9
0	0	0.58	0.90	1.12	1.24	1.32	1.41	1.45

When CR<0.1, the judgment matrix consistency test is considered to be qualified [41]. Otherwise, the judgment matrices need to be adjusted, and the consistency test is performed again until it is qualified. The consistency test results are shown in Table 15.

**Table 15:** Consistency test results.

Consistency indicators	V	A	B	C	D
*λ*_max_	4.071	3.009	2.000	3.025	3.018
CI	0.024	0.005	0	0.012	0.009
RI	0.900	0.580	0	0.580	0.580
CR	0.027	0.009	0	0.021	0.016

It can be seen from the calculation results that the CR values of each matrix are less than 0.1, which shows that the results have passed the consistency test and that the obtained weight values are reasonable.

#### Results analysis

Through the analysis and calculation of the evaluation indicators for the innovative design of tea sets, the weightage value and importance ranking of each indicator were obtained. From the data in Tables 8 to 13, it can be seen that the evaluation indicator with the highest weightage at the criterion layer is safety (0.44454), followed by cultural (0.28317), functionality (0.16509), and aesthetics (0.10721). According to the comprehensive weight ranking of indicators in the sub-criterion layer, the indicators that have a greater impact on the innovative design of tea set products are material safety and durability (0.29636), culturally monumental (0.15777), structural stability (0.14818), and traditional cultural characteristics (0.09068), easy-to-operate teapot handle (0.08898), and novel and beautiful appearance (0.07297). From the data results above, it can be concluded that people’s demand for modern Chinese tea set products is no longer limited to the need for functionality. It is shown that people are paying more attention to the cultural and experience aspects of the products. In addition to taking safety as the primary factor in the design of tea set products, integrating cultural connotations into the design of tea sets has become an important consumer demand. Associating tea sets with cultural stories can evoke people’s memories of traditional history and culture whenever they are used or viewed, thereby increasing the collection and commemorative value of the tea sets. In addition, the design of modern tea sets in China should reflect the traditional cultural content with Chinese characteristics to enhance product differentiation, alleviate the problem of tea set homogeneity, and enhance the attractiveness and market competitiveness of tea sets. Therefore, in the process of innovative tea set design, it is necessary to explore the traditional, cultural content with story-telling, extract typical cultural elements, and integrate them with the appearance, structure, and functionality of tea set products during the redesigning process. This integration aims to better meet the new market consumption demands. In terms of the experience of using the tea set, attention should be paid to the structural rationality and ease of operation of the product. With that in mind, the overall appearance should also be distinctive and aesthetically appealing.

### Design practice

#### Selection and refining of cultural inspiration

“The Classic of Mountains and Seas” is a classical ancient book from the pre-Qin period of ancient China. It is a mythological geography that contains strange and absurd stories as well as a collection of rare and natural objects [42]. The book covers various aspects of ancient myths, exotic animals, plants and trees, witchcraft, religion, history, folklore, and so on [43]. “The Classic of Mountains and Seas” has unique cultural connotation, aesthetic art and has great cultural influence in China and even in various other countries. The dreamlike mountains and landscapes, rare animals, myths, and fables in this book provides lots of story-telling containing cultural inspiration with Chinese characteristics that can be used by subsequent artistic creations and in modern cultural and creative designs [44]. “The Classic of Mountains and Seas” records the ancient sacred tree that is closely related to the tea tree. Its functions are somewhat similar to those of the tea tree, and the interoperability between the two can be found [45]. The origin of “The Classic of Mountains and Seas” and tea culture provides us with good innovative materials, and it has a large research potential in the field of modern tea set design. Through in-depth analysis and re-creation of unique cultural elements such as rare and exotic animals in the “The Classic of Mountains and Seas”, these traditional cultural elements can be applied to tea set design, which not only promotes and carries forward traditional culture but also provides new innovative directions for tea set design. Thus, “The Classic of Mountains and Seas” is selected in this study as the source of cultural inspiration for the innovative design practice of Chinese tea sets.

**Fig 3:**
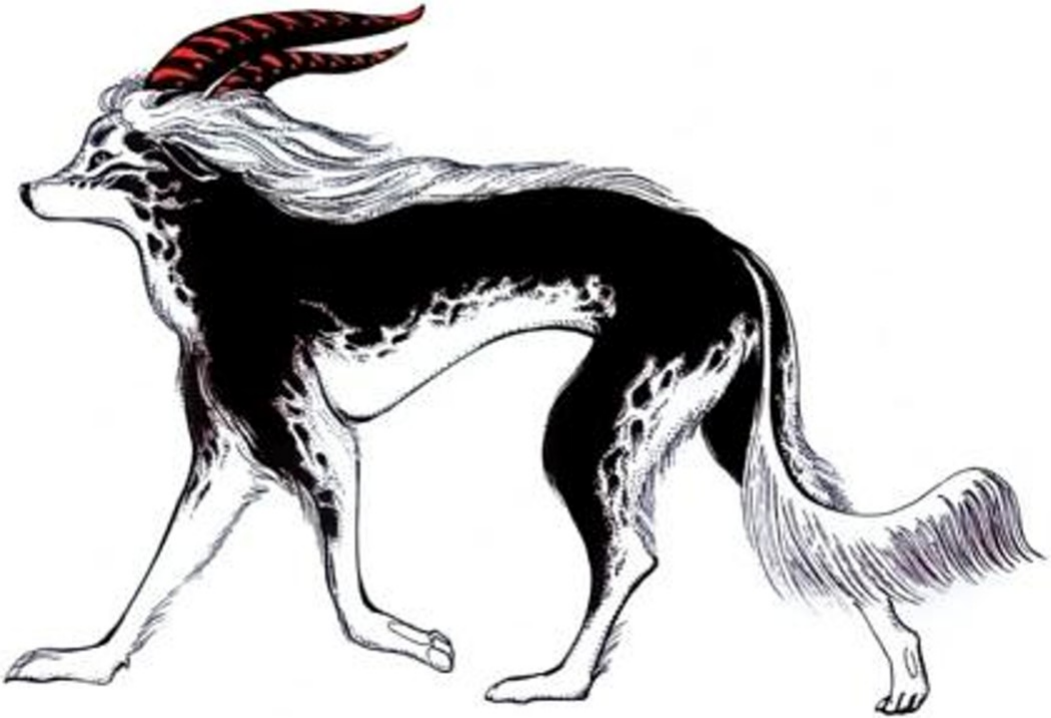
The auspicious beast—“cunning”.

It is recorded in “The Classic of Mountains and Seas”: “There is a beast in Jade Mountain. Its shape is like a dog with a leopard pattern; its horns are like an ox; its name is “Cunning”; and its sound is like a barking dog. If you see it, your country will have a good harvest.” [46] (as shown in Fig 3). The auspicious beast, “Cunning” as recorded in “The Classic of Mountains and Seas” was selected to be used in the process of developing tea set design schemes. Cultural design elements were extracted based on the appearance and behavior of the auspicious beast and applied accordingly into the design of the tea sets. According to the culture-three-level theory proposed by scholar Leong, culture can be divided into the outer “tangible” level, the middle “behavioral” level, and the inner “intangible” level [47]. Thus, the selection and extraction of cultural elements related to “Cunning” in this study are also divided into three levels: The elements at the outer “tangible” level are taken from the appearance characteristics of “Cunning”; the elements at the middle “behavioral” level are taken from the geographical environment of the auspicious beast’s behavioral activities; and the elements at the inner “intangible” level are taken from the beautiful spiritual meaning of the auspicious beast “Cunning”.

**Fig 4:**
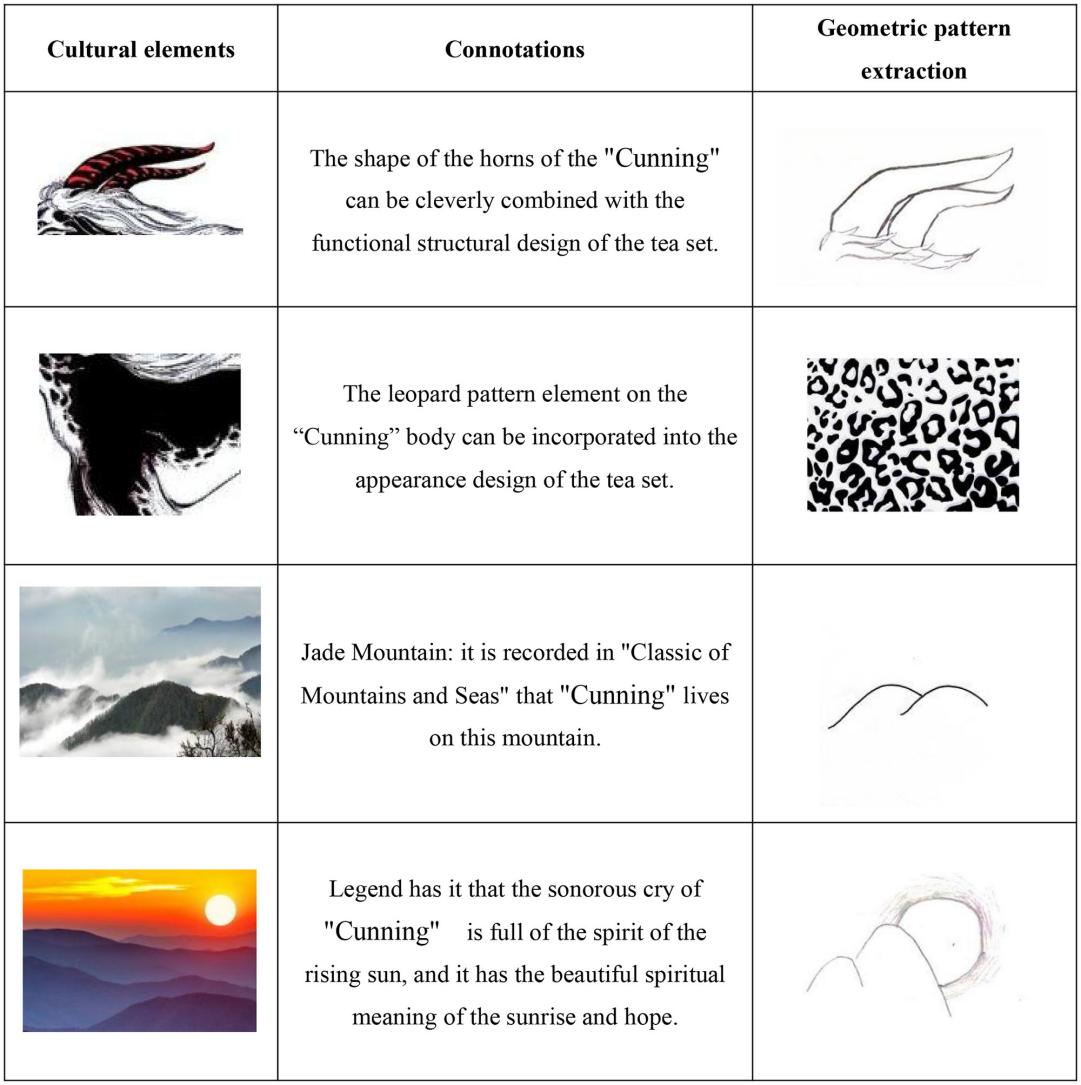
Extraction of cultural elements.

According to morphological perception and psychology in visual art, when people recognize morphology, the most prominent features are captured first. The human eye is accustomed to separating any observed object into the simplest shape allowed by known conditions [48, 49]. Therefore, this study further abstracts the cultural elements at all levels related to the auspicious beast “Cunning” into simple lines or geometric patterns and applies them to the innovative design practice of tea sets to enhance the traditional cultural characteristics and commemoration of tea sets while also increasing the innovation and interest of the appearance, making it more novel and beautiful. The extraction of cultural elements is shown in Fig 4.

#### Preliminary scheme design

**Fig 5:**
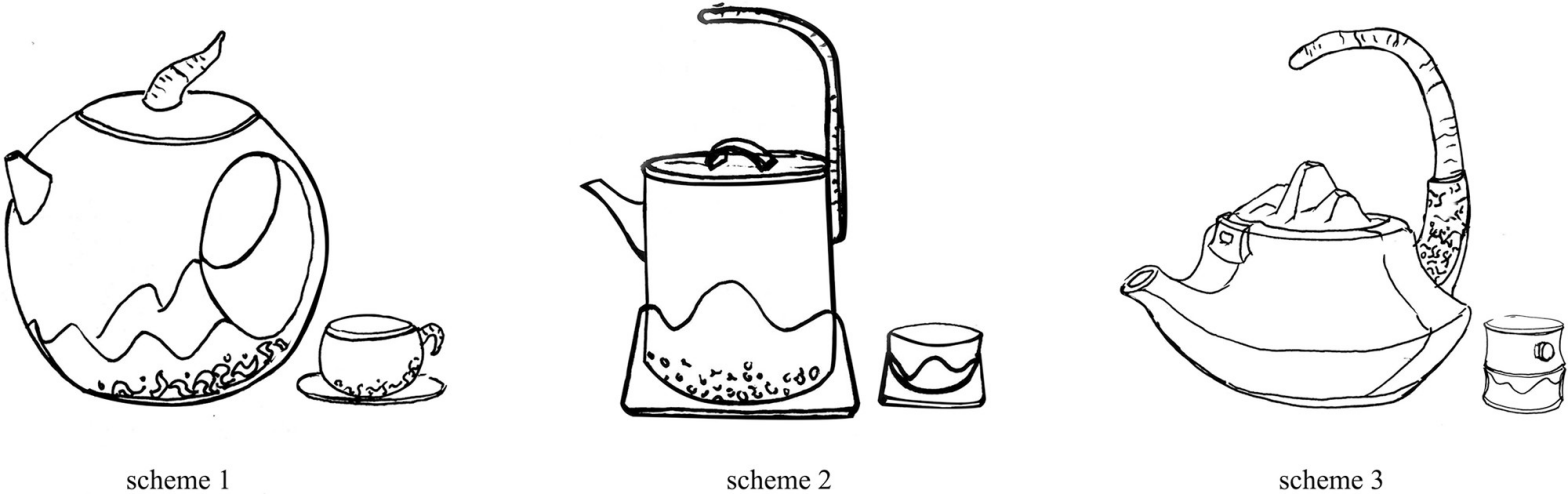
Preliminary design schemes.

Based on the weight calculation results of the indicators and the extraction of the characteristic cultural inspiration in “The Classic of Mountains and Seas,” the representative characteristic symbols and the design expectations of the tea set are skillfully integrated to carry out innovative design practices. In the innovative design of tea set products, it is necessary to consider the user experience at the visceral, behavioral, and reflective levels. The visceral level elements are mainly reflected in aesthetics, requiring a novel and appealing appearance design. The behavioral level elements focus on the safety of materials and product structures and ease of operation. On the other hand, the reflective layer elements encompass the cultural connotations and traditional characteristics embedded in the product. This study obtained three preliminary schemes by incorporating the extracted cultural elements from the “Classic of Mountains and Seas” into innovative designs at three experiential levels of tea set products, as shown in Fig 5.

1. Design scheme 1
  The material selected for this tea set scheme is sheep-fat jade porcelain, which is very durable, making it resistant to wear and deformation. This material has a fresh, elegant color with a smooth and delicate texture. The sheep-fat jade porcelain also possesses good heat preservation properties, ensuring safety and health. The overall shape of the teapot is round and full, with a solid and stable structure that prevents it from tipping over. The structural design of the top of the teapot lid and the handle of the teacup cleverly combined the shape of the horns of “Cunning” by using the natural texture and color of black sandalwood which is both aesthetically pleasing and easy to hold. The handle of the teapot is connected to the body, and the overall lines are smooth and natural, making it easy to use. The hollow circle formed by the body of the pot and the handle is next to the pattern of rolling mountains on the body of the pot. These elements create an artistic conception of the rising sun, implying vitality and blessings for good health and prosperity. The leopard print design on the bottom of the pot resembles a flowing river. The flowing river element combined with design elements of mountains, rising sun, and beast horn, makes the tea set culturally associative, and is able to evoke memories of the distinctive culture from the “Classic of Mountains and Seas”. The above adds traditional cultural characteristics to the product while also imbuing the tea set with a certain degree of collectible and commemorative value.
2. Design scheme 2
  The overall design of this scheme features simple line shapes, while the solid and stable base structure helps prevent the tea set from tipping over easily. Both the body of the teapot and teacup are made of black pottery material. The black pottery has a smooth surface that is wear-resistant, does not fade or discolor, and a dignified texture that is safe and non-toxic. The teapot handle ingeniously combines the shape of the horns of “Cunning” and is made of black sandalwood, showing the texture and color of the material itself. The spout is curved, allowing water to flow smoothly and steadily. The pattern of rolling mountains on the body of the pot and teacup is made of gradient gray combined with the texture of black pottery to create a mysterious and ethereal artistic conception, symbolizing the mystique of the Jade Mountain where the mythical beast “Cunning” lives. The irregularly distributed leopard pattern design on the bottom of the teapot body resembles a flowing river. In addition, the handle at the top of the teapot lid is semi-circular in shape, resembling the rising sun when viewed from the side.
3. Design scheme 3
  The material chosen for the body of the teapot and teacup in this scheme is white ceramic, with the teapot handle crafted from natural solid wood and black pottery for the teapot lid. These materials are safe, durable, and have a fine texture with a natural and elegant color combination. The scheme has a full overall shape, smooth lines, and a stable structure. The teapot handle which is shaped like the horn of the mythical beast “Cunning”, is designed as a semi-lifting handle. The spout of the teapot is short and tilted to ensure a smooth and powerful water flow. The design of the teapot lid combines the extracted shape of continuous mountains, making it both aesthetically pleasing and easy to use. The pattern of undulating mountains on the teacup body is made of gradient gray. There are warm orange circular solid wood handles on both sides of the cup body, echoing the pattern of the mountains, like the scene of the morning sun rising from the mountains. This reflects the spirit of vitality, thriving, and prosperity.

#### Evaluation of schemes based on the fuzzy comprehensive evaluation method

The fuzzy comprehensive evaluation method can effectively solve complex problems in design evaluation that are difficult to quantify due to fuzzy subjective judgments and have many design elements [31]. The evaluators in the index evaluation were invited to use the FCE method combined with the tea set evaluation hierarchical model to evaluate the three-tea set preliminary design schemes.

The specific process of using the fuzzy comprehensive evaluation method to assess design schemes is as follows:

1. Establish the evaluation indicator set and use the criterion layer evaluation indicators as the factor set V, *V* = {*V*_*A*_, *V*_*B*_, *V*_*C*_, V_*D*_}. The factor set of the sub-criteria layer index is expressed as a_*i*_ = {*a*_1_, *a*_2_,…, *a*_*n*_}(*i* = 1, 2, 3).
2. Using Likert’s five-point scale to determine the evaluation levels and their corresponding scoring criteria. Create the evaluation set *X* = {*X*_1_, *X*_2_, *X*_3_, *X*_4_} = {very satisfied, satisfied, generally satisfied, dissatisfied, very dissatisfied}, and assign different evaluation scores to different evaluation levels. The evaluation levels in the evaluation set respectively correspond to the evaluation scores: 90–100, 80–90, 70–80, 60–70, below 60.
3. Construction of the fuzzy comprehensive evaluation matrix. The expert group evaluators were invited to assess the three preliminary design schemes using the tea set evaluation hierarchy model. The times of scores given by evaluators are counted, and the degree of membership of each evaluation indicator relative to each evaluation level is obtained. Thus, the fuzzy comprehensive evaluation matrix R of each indicator of each tea set design scheme is constructed. Taking Scheme 1 as an example, *R*_*A*_ represents the evaluation matrix of the criterion layer functionality for Scheme 1; *R*_*B*_ represents the evaluation matrix of the criterion layer safety for Scheme 1; R C represents the evaluation matrix of the criterion layer aesthetic for Scheme 1; and *R*_*D*_ represents the evaluation matrix of the criterion layer cultural for Scheme 1:
  $$\begin{array}{c} R_{A}=[\begin{array}{ccccc} 0.2 & 0.5 & 0.3 & 0 & 0\\ 0.3 & 0.6 & 0.1 & 0 & 0\\ 0.1 & 0.6 & 0.3 & 0 & 0 \end{array}] \end{array}$$
  $$\begin{array}{c} R_{B}=[\begin{array}{ccccc} 0.7 & 0.3 & 0 & 0 & 0\\ 0.5 & 0.4 & 0.1 & 0 & 0 \end{array}] \end{array}$$
  $$\begin{array}{c} R_{C}=[\begin{array}{ccccc} 0.3 & 0.6 & 0.1 & 0 & 0\\ 0.4 & 0.6 & 0 & 0 & 0\\ 0.2 & 0.7 & 0.1 & 0 & 0 \end{array}] \end{array}$$
  $$\begin{array}{c} R_{D}=[\begin{array}{ccccc} 0.2 & 0.6 & 0.2 & 0 & 0\\ 0.5 & 0.4 & 0.1 & 0 & 0\\ 0.4 & 0.5 & 0.1 & 0 & 0 \end{array}] \end{array}$$
4. To better comprehensively utilize the information derived from indicator weights and the fuzzy comprehensive evaluation matrix, a weighted average type fuzzy operator is employed to mathematically synthesize the weights of each evaluation indicator with their corresponding evaluation matrix R. This process calculates the evaluation weight vectors P for each indicator in the criterion layer of the tea set scheme 1.
  According to Tables 9 to 12, the weight values of the indicators were obtained:
  $$\begin{array}{c} \omega_{A}=\left(0.16378\;0.53896\;0.29726\right) \end{array}$$
  $$\begin{array}{c} \omega_{B}=\left(0.66667\;0.33333\right) \end{array}$$
  $$\begin{array}{c} \omega_{C}=\left(0.20141\;0.11795\;0.68064\right) \end{array}$$
  $$\begin{array}{c} \omega_{D}=\left(0.12262\;0.32024\;0.55714\right) \end{array}$$
  The weight vectors of the criterion layer indicators of the tea set design scheme can be calculated:
  $$\begin{array}{c} P_{A}=\omega_{A}\circ R_{A}=\left(0.224\;0.584\;0.192\;0.000\;0.000\right) \end{array}$$
  $$\begin{array}{c} P_{B}=\omega_{B}\circ R_{B}=\left(0.633\;0.333\;0.033\;0.000\;0.000\right) \end{array}$$
  $$\begin{array}{c} P_{C}=\omega_{C}\circ R_{C}=\left(0.244\;0.668\;0.088\;0.000\;0.000\right) \end{array}$$
  $$\begin{array}{c} P_{D}=\omega_{D}\circ R_{D}=\left(0.408\;0.480\;0.112\;0.000\;0.000\right) \end{array}$$
5. On this basis, the fuzzy comprehensive evaluation matrix for the target layer can be constructed:
  $$\begin{array}{c} P_{V}=[\begin{array}{c} P_{A}\\ P_{B}\\ P_{C}\\ P_{D} \end{array}]=[\begin{array}{ccccc} 0.224 & 0.584 & 0.192 & 0.000 & 0.000\\ 0.633 & 0.333 & 0.033 & 0.000 & 0.000\\ 0.244 & 0.668 & 0.088 & 0.000 & 0.000\\ 0.408 & 0.480 & 0.112 & 0.000 & 0.000 \end{array}] \end{array}$$
6. From the above, the comprehensive evaluation vector of the tea set design scheme can be obtained:
  $$\begin{array}{c} S=\omega_{V}\circ P_{V}=\left(0.460\;0.452\;0.088\;0\;0\right) \end{array}$$

The calculation shows that the total evaluation score of the tea set innovative design scheme 1 is N = 83.72. Similarly, it can be concluded that the score of scheme 2 is 77.82 and the score of scheme 3 is 80.62. Based on the above scores, it is evident that scheme 1 is the optimal design scheme. (The calculation data for schemes 2 and 3 can be found in the attachment S1 File.).

**Fig 6:**
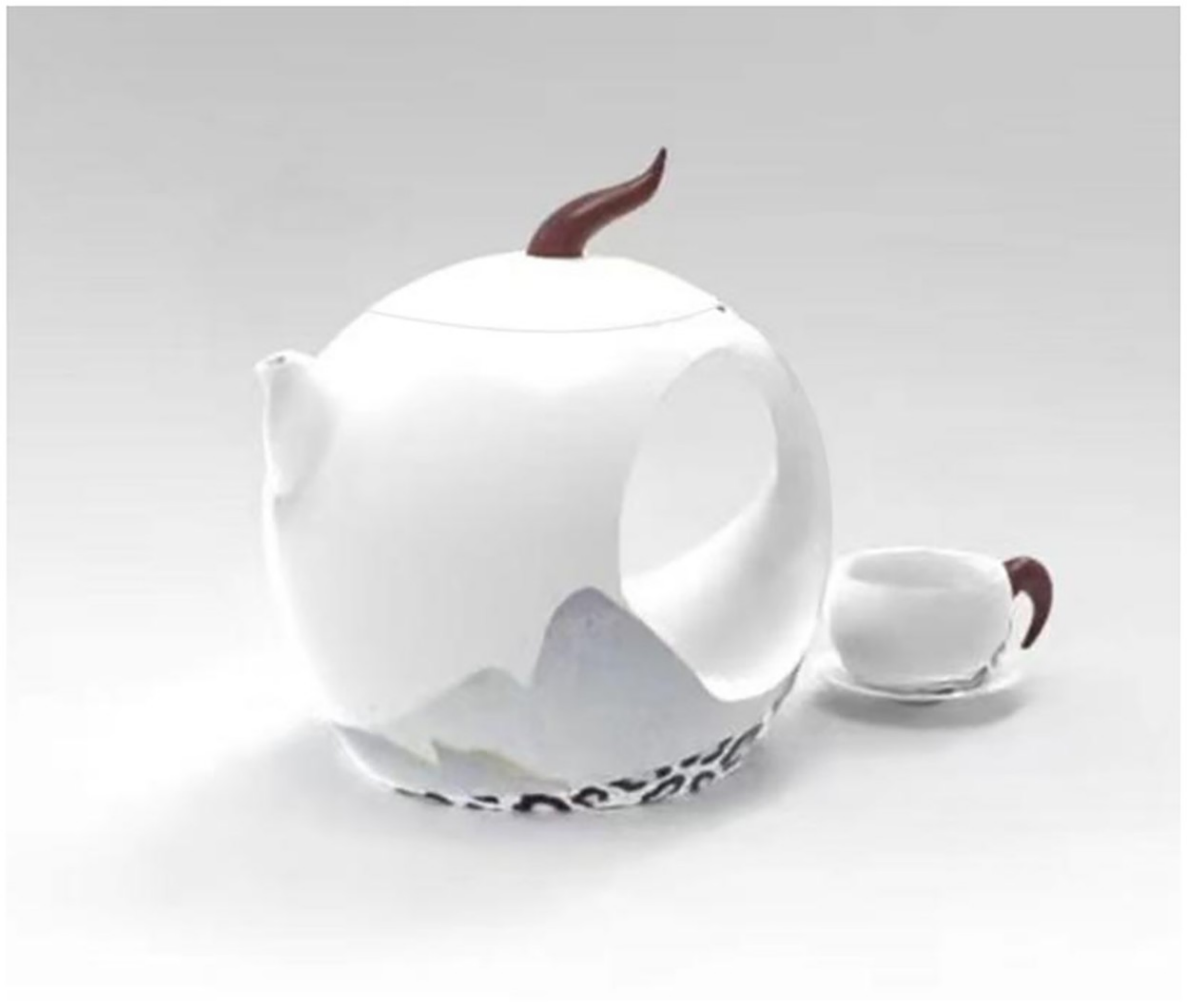
Tea set innovative design scheme rendering.

Subsequently, Scheme 1 is refined and designed based on the evaluation results. The evaluation results indicate that the rating result of the tea set innovative design scheme 1 belongs to the “satisfied” level, indicating that the scheme overall meets consumer demands. Analysis of the evaluation scores for various indicators of this scheme reveals that the score for the “discharge water smoothly and steadily, fully filtering the tea residue” indicator is relatively low, suggesting potential for optimization in this preliminary scheme. Discussions among the researchers analyzed the reasons for the low scores in these two evaluation indicators. This could be attributed to the insufficiently reasonable design of the spout of the teapot. Consequently, in the final scheme, optimizations were made to the spout of the teapot design by adjusting the angle and form of the spout, making it short, tilted, curved, and with a certain degree of protrusion, thereby ensuring smooth and powerful water outflow while effectively preventing the pouring out of tea residues to a certain extent. The final scheme rendering is shown in Fig 6.

## Discussion

With regards to the research on modern tea sets, it is found that many scholars currently pay more attention to the research on the application methods of modern technology in the development of tea sets. However, it is also found that there are very few studies on the design evaluation of modern tea set products. Developing a comprehensive tea set product evaluation method can not only help designers more efficiently design product solutions that better meet new consumer needs but also evaluate design solutions more objectively and rationally in order to continuously optimize and improve the solutions. Based on the grounded theory, this study firstly constructed a hierarchical model of evaluation indicators for tea set products. Next, the analytical hierarchy process was used to calculate the weight values and ranked the evaluation indicators according to importance to develop a design guideline for the subsequent design activity. Finally, a comprehensive evaluation of the design plan through the fuzzy comprehensive evaluation method was conducted to help optimize the scheme. However, this study still has certain limitations. First and foremost, the number of expert samples for interviews and index scoring was relatively small. Although the use of grounded theory can reduce the subjectivity of the evaluation index determination process, due to the relatively single calculation method and the limited number of expert groups, the results obtained still have certain limitations. In subsequent research, the number of experts interviewed and evaluated can be further expanded, and a variety of calculation methods can be combined to make the evaluation more complete. Secondly, this article does not classify the interviewees in detail. People in different regions or working in different jobs may have different understandings of the same product, and the results obtained may also be affected. Therefore, this study can conduct more targeted research on people in different regions and different types of work in the future.

## Conclusion

This study conducted a systematic coding analysis of the interview data through grounded theory, thereby largely avoiding subjectivity in the process of summarizing evaluation indicators and constructing a hierarchical model for tea set product evaluation in a more scientific and logical manner. Subsequently, utilizing the Analytic Hierarchy Process (AHP), this study calculated the weightage and importance priorities of evaluation indicators at each level. This method simultaneously incorporates the demand indicator weights rankings and demand hierarchies into the product design positioning decision-making process, which reduces design positioning bias. Furthermore, the innovative design practice of tea sets was carried out based on the unique cultural elements from “The Classic of Mountains and Seas.”. Finally, the fuzzy comprehensive evaluation method was used to select the optimal design scheme. The methodological framework proposed in this study comprehensively utilizes the methodological advantages of grounded theory, the analytic hierarchy process, and the fuzzy comprehensive evaluation method. It can provide scientific methodological support for multiple phases in the entire tea set design process, thus helping the design process become clearer and more systematic. Compared to purely using the perceptual experience method, the research results obtained from this integrated approach are more scientific and rigorous. Unlike other single-design methods, the combined approach proposed in this study more comprehensively considers the qualitative and quantitative factors in tea set product design, reducing the influence of the subjectivity of decision-makers and ensuring the design and evaluation are more comprehensive, accurate, and rational. The GT-AHP-FCE method framework can provide designers with specific and feasible design guidance and decision support, helping them better analyze the diverse demands of consumers to design more competitive and appealing tea set products. By ingeniously incorporating traditional cultural resources into tea set innovation design, this research will be able to contribute to the development of the tea set industry and the inheritance and innovation of Chinese traditional culture.

## Supporting information

S1 File Calculate data supplements. (DOCX)

S1 Appendix Informed consent form. (DOCX)

S2 Appendix Interview content. (DOCX)
