# Supplementary material for: Research on an innovative design and evaluation method of Chinese tea sets based on GT-AHP-FCE
Source: PLoS One. 2024 Apr 11;19(4):e0302005. doi: 10.1371/journal.pone.0302005 (PMC11008883; doi:10.1371/journal.pone.0302005)
Supplement: S1 File — (DOCX) [file pone.0302005.s001.docx]

The following is the specific process of calculating the final evaluation scores of Schemes 2 and 3 in Chapters 3.3.2 (Fig. 4) and 3.3.3 through the fuzzy comprehensive evaluation method:

**1. Scheme 1**

Count the number of times evaluators score each indicator in the sub-criterion layer, and obtain the degree of membership of each evaluation indicator relative to each evaluation level. Thus, the fuzzy comprehensive evaluation matrix R of each indicator of tea set design scheme 2 is constructed.$R_{A}$represents the evaluation matrix of the criterion layer functionality for Scheme 2; $R_{B}$represents the evaluation matrix of the criterion layer safety for Scheme 2; $R_{C}$represents the evaluation matrix of the criterion layer aesthetic for Scheme 2; and $R_{D}$represents the evaluation matrix of the criterion layer cultural for Scheme 2:

$$R_{A}=\left[ \begin{matrix} 0.1 & 0.5 & 0.3 & 0.1 & 0 \\ 0 & 0.3 & 0.3 & 0.4 & 0 \\ 0.1 & 0.4 & 0.5 & 0 & 0 \end{matrix} \right]$$

$$R_{B}=\left[ \begin{matrix} 0.1 & 0.7 & 0.2 & 0 & 0 \\ 0.5 & 0.4 & 0.1 & 0 & 0 \end{matrix} \right]$$

$$R_{C}=\left[ \begin{matrix} 0.1 & 0.5 & 0.4 & 0 & 0 \\ 0.1 & 0.6 & 0.3 & 0 & 0 \\ 0 & 0.5 & 0.5 & 0 & 0 \end{matrix} \right]$$

$$R_{D}=\left[ \begin{matrix} 0.1 & 0.5 & 0.4 & 0 & 0 \\ 0.3 & 0.4 & 0.3 & 0 & 0 \\ 0.1 & 0.4 & 0.5 & 0 & 0 \end{matrix} \right]$$

(4) Using a weighted average type fuzzy operator to synthesize the weights of each indicator with their corresponding evaluation matrix R, the evaluation weight vectors P for each indicator in the criterion layer of Scheme 2 is calculated.

According to Tables 9–12, the weight values of the indicators were obtained:

$$\omega_{A}=\left( 0.16378 0.53896 0.29726 \right)$$

$$\omega_{B}=\left( 0.66667 0.33333 \right)$$

$$\omega_{C}=\left( 0.20141 0.11795 0.68064 \right)$$

$$\omega_{D}=\left( 0.12262 0.32024 0.55714 \right)$$

The weight vectors of the criterion layer indicators of the design scheme 2 can be calculated:

$$P_{A}=\omega_{A}\circ R_{A}=\left( 0.046 0.362 0.359 0.232 0.000 \right)$$

$$P_{B}=\omega_{B}\circ R_{B}=\left( 0.233 0.600 0.167 0.000 0.000 \right)$$

$$P_{C}=\omega_{C}\circ R_{C}=\left( 0.032 0.512 0.456 0.000 0.000 \right)$$

$$P_{D}=\omega_{D}\circ R_{D}=\left( 0.164 0.412 0.424 0.000 0.000 \right)$$

1. On this basis, the fuzzy comprehensive evaluation matrix for the target layer can be constructed:

$$P_{V}=\left[ \begin{matrix} P_{A} \\ P_{B} \\ P_{C} \\ P_{D} \end{matrix} \right]=\left[ \begin{matrix} 0.046 & 0362 & 0.359 & 0.232 & 0.000 \\ 0.233 & 0.600 & 0.167 & 0.000 & 0.000 \\ 0.032 & 0.512 & 0.456 & 0.000 & 0.000 \\ 0.164 & 0.412 & 0.424 & 0.000 & 0.000 \end{matrix} \right]$$

1. From the above, the comprehensive evaluation vector of the tea set design scheme 2 can be obtained:

$$S=\omega_{V}\circ P_{V}=\left( 0.161 0.498 0.303 0.038 0 \right)$$

The calculation shows that the total evaluation score of the tea set innovative design scheme 2 is N = 77.82.

**2. Scheme 3**

Count the number of times evaluators score each indicator in the sub-criterion layer, and obtain the degree of membership of each evaluation indicator relative to each evaluation level. Thus, the fuzzy comprehensive evaluation matrix R of each indicator of tea set design scheme 3 is constructed.$R_{A}$represents the evaluation matrix of the criterion layer functionality for Scheme 3; $R_{B}$represents the evaluation matrix of the criterion layer safety for Scheme 3; $R_{C}$represents the evaluation matrix of the criterion layer aesthetic for Scheme 3; and $R_{D}$represents the evaluation matrix of the criterion layer cultural for Scheme 3:

$$R_{A}=\left[ \begin{matrix} 0.2 & 0.6 & 0.2 & 0 & 0 \\ 0 & 0.3 & 0.6 & 0.1 & 0 \\ 0.1 & 0.4 & 0.5 & 0 & 0 \end{matrix} \right]$$

$$R_{B}=\left[ \begin{matrix} 0.6 & 0.4 & 0 & 0 & 0 \\ 0.4 & 0.5 & 0.1 & 0 & 0 \end{matrix} \right]$$

$$R_{C}=\left[ \begin{matrix} 0 & 0.6 & 0.4 & 0 & 0 \\ 0.1 & 0.6 & 0.3 & 0 & 0 \\ 0.1 & 0.6 & 0.3 & 0 & 0 \end{matrix} \right]$$

$$R_{D}=\left[ \begin{matrix} 0.1 & 0.5 & 0.4 & 0 & 0 \\ 0.3 & 0.5 & 0.2 & 0 & 0 \\ 0.1 & 0.6 & 0.3 & 0 & 0 \end{matrix} \right]$$

(4) Using a weighted average type fuzzy operator to synthesize the weights of each indicator with their corresponding evaluation matrix R, the evaluation weight vectors P for each indicator in the criterion layer of Scheme 3 is calculated.

According to Tables 9–12, the weight values of the indicators were obtained:

$$\omega_{A}=\left( 0.16378 0.53896 0.29726 \right)$$

$$\omega_{B}=\left( 0.66667 0.33333 \right)$$

$$\omega_{C}=\left( 0.20141 0.11795 0.68064 \right)$$

$$\omega_{D}=\left( 0.12262 0.32024 0.55714 \right)$$

The weight vectors of the criterion layer indicators of the design scheme 3 can be calculated:

$$P_{A}=\omega_{A}\circ R_{A}=\left( 0.062 0.379 0.505 0.054 0.000 \right)$$

$$P_{B}=\omega_{B}\circ R_{B}=\left( 0.533 0.433 0.033 0.000 0.000 \right)$$

$$P_{C}=\omega_{C}\circ R_{C}=\left( 0.080 0.600 0.320 0.000 0.000 \right)$$

$$P_{D}=\omega_{D}\circ R_{D}=\left( 0.164 0.556 0.280 0.000 0.000 \right)$$

1. On this basis, the fuzzy comprehensive evaluation matrix for the target layer can be constructed:

$$P_{V}=\left[ \begin{matrix} P_{A} \\ P_{B} \\ P_{C} \\ P_{D} \end{matrix} \right]=\left[ \begin{matrix} 0.062 & 0379 & 0.505 & 0.054 & 0.000 \\ 0.533 & 0.433 & 0.033 & 0.000 & 0.000 \\ 0.080 & 0.600 & 0.320 & 0.000 & 0.000 \\ 0.164 & 0.556 & 0.280 & 0.000 & 0.000 \end{matrix} \right]$$

1. From the above, the comprehensive evaluation vector of the tea set design scheme 3 can be obtained:

$$S=\omega_{V}\circ P_{V}=\left( 0.302 0.477 0.212 0.009 0 \right)$$

The calculation shows that the total evaluation score of the tea set innovative design scheme 3 is N = 80.62.
