# Supplementary figures and images for: Research on an innovative design and evaluation method of Chinese tea sets based on GT-AHP-FCE
Source: PLoS One. 2024 Apr 11;19(4):e0302005. doi: 10.1371/journal.pone.0302005 (PMC11008883; doi:10.1371/journal.pone.0302005)

Below are the informed consent forms signed by the respondents.


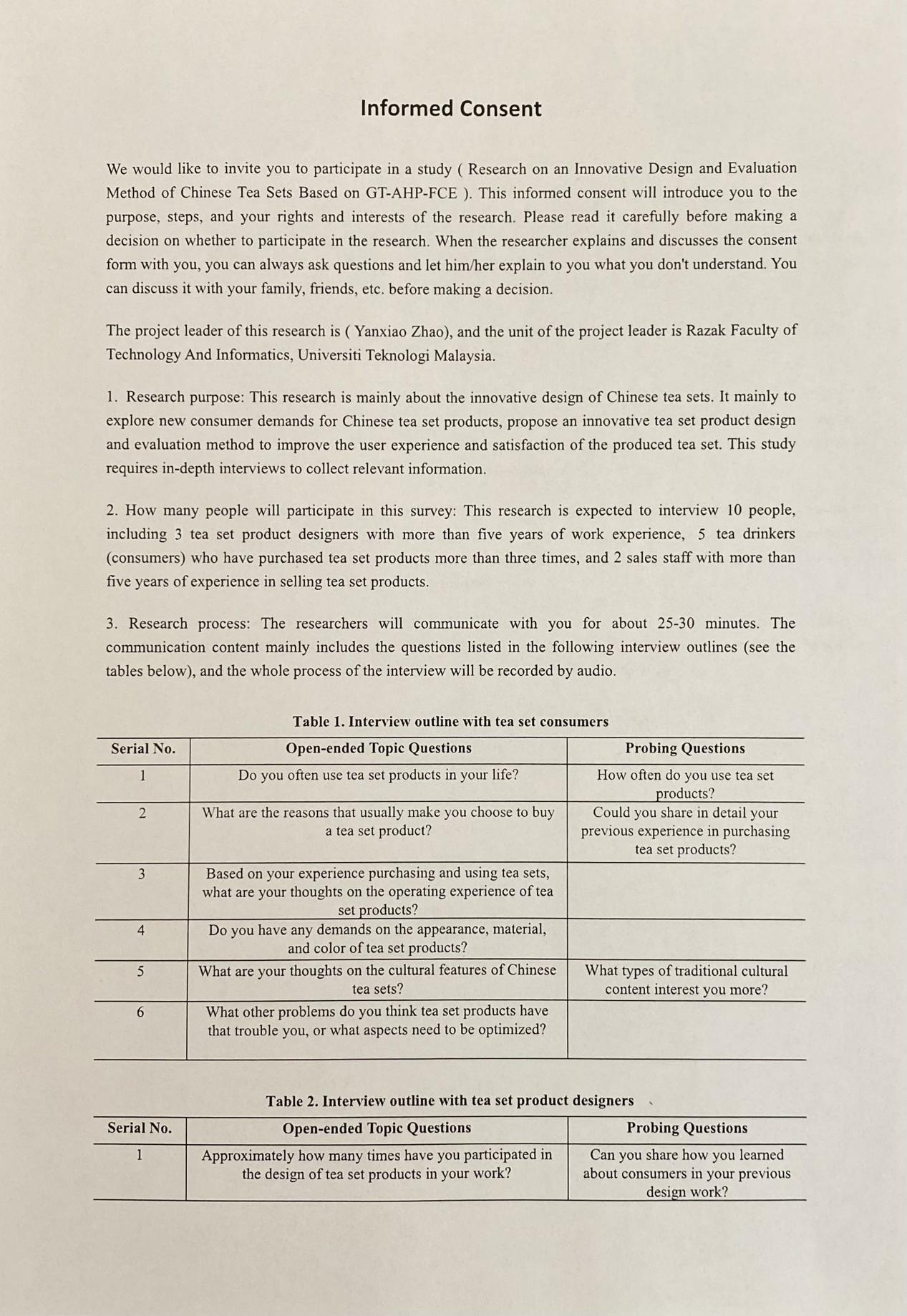


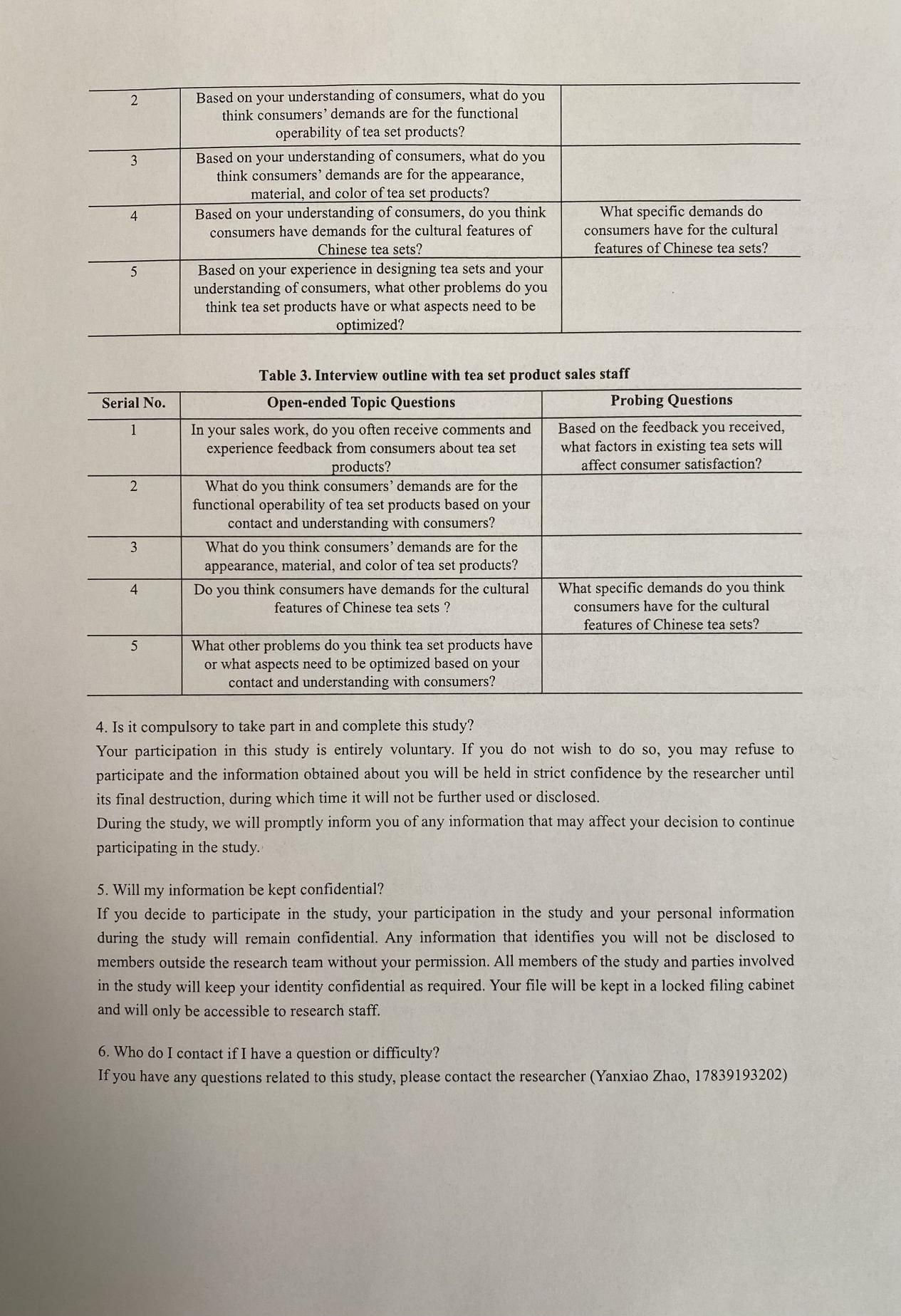


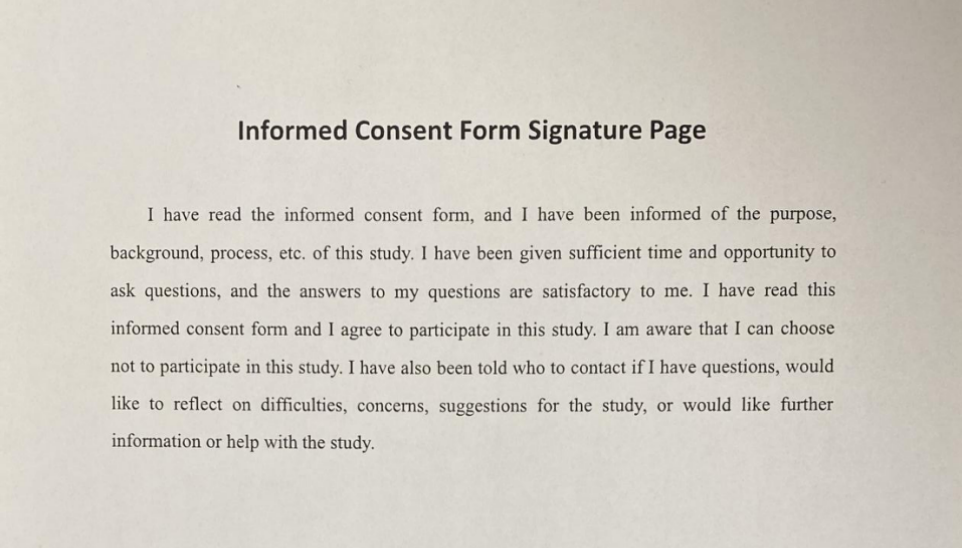

Supplement: S1 Appendix — (DOCX) [file pone.0302005.s002.docx]
