# Supplementary material for: Research on an innovative design and evaluation method of Chinese tea sets based on GT-AHP-FCE
Source: PLoS One. 2024 Apr 11;19(4):e0302005. doi: 10.1371/journal.pone.0302005 (PMC11008883; doi:10.1371/journal.pone.0302005)
Supplement: S2 Appendix — (DOCX) [file pone.0302005.s003.docx]

**Content of the interview**

The respondents of this study involved two experienced tea set product designers, one professor in the field of Chinese tea culture research who is also a tea set product designer, five consumers who have purchased tea set products more than three times, and two sales staff with years of experience in tea set product sales.

| **Basic information of respondent** | | | |
| --- | --- | --- | --- |
| Identity of the respondent | Tea set product designer 1 | Gender | female |
| Years of relevant work experience | 11 years | Education Qualifications | graduate degree |
| Place of interview | online | | |
| **Summary and record of interview key points** | | | |
| I have participated in and led the development of about a dozen tea set product design projects. In the first two or three years of my work, I was responsible for a lot of user research work. In previous design work, I usually understood consumers through market research and consumer feedback on previous tea set products. Consumers' requirements for the functional operation of tea set products are mainly ease of use, such as whether the tea set is easy to pick up and put down, whether the teapot and teacup are easy to hold and not hot when used, whether the water from the teapot spout is smooth, or whether the water is discharged too quickly, causing it to splash easily. Everyone may have different preferences regarding the appearance, material, and color of tea set products, which are related to personal aesthetics. However, in general, tea-loving consumers prefer simple and plain color matching, and the appearance should be novel and beautiful. The material is safe and wear-resistant. In recent years, China has increasingly promoted cultural innovation, and consumers' demand for cultural experiences and cultural features in products is also rising. Chinese traditional cultural characteristics can empower tea set products. | | | |

| **Basic information of respondent** | | | |
| --- | --- | --- | --- |
| Identity of the respondent | Tea set product designer 2 | Gender | male |
| Years of relevant work experience | 17 years | Education Qualifications | undergraduate |
| Place of interview | online | | |
| **Summary and record of interview key points** | | | |
| I have participated in about 20 to 30 large and small tea set product design projects, of which fifteen tea set projects were led by me. We usually conduct consumer research in the early stages of a project. Our company has online sales platform stores, so we also learn about consumers’ thoughts from product reviews in online stores. The most basic consumer demand for tea set products is the practicality and convenience of functional operations. Specifically, it is convenient to filter tea leaves, pour and add water, save labor, and not burn hands. The appearance, material, and color of tea set products need to bring visual enjoyment to users. Consumers generally prefer neutral tones for the color matching of tea set products. More and more young people are beginning to like drinking tea, and they require more innovative and interesting tea sets. The material selection should have good texture and durability. China has a long history of tea culture and is also rich in other cultural resources. However, many existing Chinese tea sets do not reflect the characteristics of its long-standing traditional culture and lack distinction from products from other countries. In recent years, consumers have also become very fond of tea sets with stories and culture, and some people even buy them just for collection. Therefore, appropriately integrating elements and symbols that reflect traditional cultural content with Chinese characteristics is also a trend in current tea set design. | | | |

| **Basic information of respondent** | | | |
| --- | --- | --- | --- |
| Identity of the respondent | Professor in the field of Chinese tea culture research and concurrently serving as tea set product designer | Gender | male |
| Years of relevant work experience | 24 years | Education Qualifications | Doctoral degree |
| Place of interview | online | | |
| **Summary and record of interview key points** | | | |
| In the course of studying Chinese tea culture, investigation and exploration of tea sets are indispensable. I have been involved to varying extents in approximately ten tea set design projects. Our interaction with consumers and understanding of them primarily relied on research into consumer behavior and market sales data in the process of these design projects. In recent years, the country has vigorously supported and promoted cultural innovation, and people's living standards have continued to improve, so people have paid more and more attention to the quality of life, and tea drinking culture has once again set off a craze. Consumers' demand for tea set products is not only to meet functional requirements but also to value the tea drinking experience and spiritual enjoyment brought by the cultural artistic conception of tea sets. Therefore, this also puts forward more diverse new requirements for the current tea set product development. Consumers have the most basic needs for the functions of tea set products, including: the operation process is simple and convenient; the tea leaves and tea water can be well separated; the teapot lid does not buckle unstablely; or the teacup has no handle, which makes your hands hot when drinking tea. Since the consumer group of tea set products is showing a younger trend, the appearance design of tea set products can no longer be so serious and dull but should appropriately increase the fashion sense and fun of the appearance. Consumers’ color preference for tea set products is Zen-like neutral tones. In addition, many consumers report that sometimes they want to update the tea sets at home when they see a tea set they like. However, it is a pity to throw away the old tea set after buying a new tea set, but it has no collection value or commemorative value worthy of me not throwing it away. Therefore, I think that if we can give tea sets collection and commemorative value by incorporating storytelling cultural content, this problem of consumers may be effectively solved. | | | |

| **Basic information of respondent** | | | |
| --- | --- | --- | --- |
| Respondent persona | Tea set consumer 1 | Age | 46 |
| Gender | male | Education Qualifications | Undergraduate |
| Frequency of using tea set | average two to three times a week | Place of interview | online |
| **Summary and record of interview key points** | | | |
| I use tea sets quite frequently. I like to drink tea with others at home or when I go out to discuss business. On average, I use tea products two to three times a week. I like to study tea sets and tea culture, so when I choose to buy tea sets, I pay great attention to their use experience. Of course, the novel and unique appearance of the tea set will also attract me to buy it. Regarding some of the tea sets I have used, some of them are very uncomfortable to operate. For example, the handles of some teapots are designed on the top of the teapot, which makes pouring tea very laborious. I like tea sets that can reflect some Chinese characteristics in appearance, because now many Chinese tea sets are indistinguishable from Japanese or other countries’ tea sets. I quite like the content of traditional Chinese culture, it would be better if I could see it in the design of tea sets. Regarding the material of the tea set, I think it should first be healthy and non-toxic, and it is best to be able to retain heat. In addition, I have several tea sets at home. Every time I see a tea set that is novel in appearance and easy to use, I can’t help but buy one. After I buy new tea sets, the old tea sets that I replace at home are usually rarely used, but most of them have no collection value or commemorative significance, so there is no need to keep them at home, but throwing them away would feel a bit wasteful. | | | |

| **Basic information of respondent** | | | |
| --- | --- | --- | --- |
| Respondent persona | Tea set consumer 2 | Age | 40 |
| Gender | male | Education Qualifications | graduate degree |
| Frequency of using tea set | Average once a week | Place of interview | online |
| **Summary and record of interview key points** | | | |
| I often use the tea set, and when I have guests over at home, I will drink tea and chat with them. On average,I use tea sets almost once a week. I like to buy some unique tea sets as gifts for my friends. I usually buy a tea set because it looks good, has good quality, or has some special features. Regarding some of the tea sets I have used, I think they are both good and bad in terms of usage experience. For example, some teapots are easily knocked over accidentally when pouring tea, and the tea burns your hands; sometimes the tea leaves cannot be filtered well, and there will be tea leaves in the tea. I don't like this very much. I like tea sets that have a stylish and simple appearance, and the material is preferably smooth and not easy to fade or stain. The color is elegant and refreshing. It is best for tea sets to reflect the cultural characteristics of different regions in China, because I used to go to local tea set stores when traveling to some places, but many tea sets looked very similar, so I was not very interested in buying them. | | | |

| **Basic information of respondent** | | | |
| --- | --- | --- | --- |
| Respondent persona | Tea set consumer 3 | Age | 58 |
| Gender | female | Education Qualifications | undergraduate |
| Frequency of using tea set | Once a day | Place of interview | online |
| **Summary and record of interview key points** | | | |
| Now I use tea products almost every day. I like to drink tea and listen to music at home after my husband and I wake up from a nap. When I choose to buy tea sets, I usually want to buy them when I see something good-looking when shopping or traveling. I like tea sets with simple color schemes and affinity materials. The colors of some tea sets are very bright and eye-catching, which is not only uncomfortable to look at but also does not match the atmosphere of tea drinking. A tea set I bought before always caused tea to splash out easily when I poured tea, which made it very unpleasant to use. I think the most basic thing about the materials of tea sets is that they should be safe and healthy and should not contain harmful substances. I love the smooth ceramic material. The round and full shape look more solid and durable. As for the cultural characteristics reflected in the tea sets, I hope to combine them with some well-known museum cultural relics, because many existing Chinese tea sets do not reflect the characteristics of China's long-standing traditional culture, and museum cultural relics have a certain degree of representativeness of traditional culture. | | | |

| **Basic information of respondent** | | | |
| --- | --- | --- | --- |
| Respondent persona | Tea set consumer 4 | Age | 25 |
| Gender | male | Education Qualifications | high school degree |
| Frequency of using tea set | once a month | Place of interview | online |
| **Summary and record of interview key points** | | | |
| I occasionally use tea set products. I go home to visit my parents once a month after I start working, and every time I go back to my parents’ house, I drink tea and chat with my parents after dinner. I have purchased tea set products as gifts for relatives and friends. When drinking tea with my parents, I am usually responsible for making and pouring the tea, so I have some experience operating the tea set. Some teacups are designed without handles, making it inconvenient to lift the teacup to drink tea if the tea is too hot. In addition, the spout of the teapot is sometimes half-blocked by incompletely filtered tea leaves, resulting in poor water flow. Many tea sets look old-fashioned, not simple and fashionable enough, and are not good-looking. I think the appearance of the tea set should be more original and unique. Regarding cultural characteristics, I think it would be better if it could reflect Chinese culture. | | | |

| **Basic information of respondent** | | | |
| --- | --- | --- | --- |
| Respondent persona | Tea set consumer 5 | Age | 33 |
| Gender | female | Education Qualifications | Doctoral degree |
| Frequency of using tea set | Average once a week | Place of interview | online |
| **Summary and record of interview key points** | | | |
| I use tea set products almost once a week on average. Every weekend, I like to drink tea and read a book on the balcony alone. I like to buy some very special tea sets. For example, when I visited the Forbidden City Museum, I bought a tea set from the cultural and creative store that has the cultural characteristics of the Forbidden City. It is very meaningful to use it myself or give it to friends. Through some of the tea set products that I have used and seen when visiting tea set stores, I feel that many of them are very similar. Many tea set shapes do not look unique or interesting and are not attractive to people like us. In terms of functional operation, I think the teapot lid, handle, and teacup handle should be easy to hold and use. The color combination of sober and simple for tea sets should be more in line with the ancient tea culture. I prefer tea set materials with texture and affinity without looking cold and stiff. Nowadays, China vigorously advocates cultural inheritance and development, but in many existing Chinese tea sets, we rarely see symbols or patterns that reflect traditional culture, and it is difficult to see what cultural connotations they contain. Therefore, if tea set products have cultural characteristics, they will also be a plus point for the product. | | | |

| **Basic information of respondent** | | | |
| --- | --- | --- | --- |
| Identity of the respondent | Tea set product sales staff 1 | Gender | female |
| Years of relevant work experience | 13 years | Education Qualifications | undergraduate |
| Place of interview | Offline: face to face | | |
| **Summary and record of interview key points** | | | |
| As sales staff, we often come into contact with various customers, and we also receive some feedback from consumers on tea sets and pre-purchase consultation. Consumers usually pay more attention to the practicality of tea set products, whether the appearance meets aesthetic requirements, and whether a tea has any distinctive features or selling points. In some feedback we received from consumers, consumers reported that some tea set products did not completely filter the tea leaves, which affected the taste of tea and easily blocked the teapot outlet. In addition, some teapot lids are not securely buckled, causing water to leak from the lid or even the teapot lid to fall off when pouring tea. Regarding the appearance of tea sets, some customers feel that many tea sets look old-fashioned, dull, and boring, have not kept pace with the times, and are easily eliminated. Consumers prefer tea set materials that look textured and are resistant to wear, fading, or discoloration. In terms of color matching, it is best to use classic and versatile colors such as black, white, and gray. In today's era that focuses on spiritual experience and cultural consumption, consumers' demands for tea set products have certainly become more diverse. Cultural characteristics are also receiving more and more attention from customers. China is rich in historical and cultural resources. Traditional customs, intangible cultural heritage, and museum relics are very popular. Consumers also like some tea sets with historical stories and cultural connotations, such as "Thousand Miles of Rivers and Mountains." A tea set designed with cultural inspiration from the museum's famous painting collection is very popular among consumers. Therefore, we expect that Chinese tea sets can better reflect unique cultural characteristics and enhance their appeal to consumers. | | | |

| **Basic information of respondent** | | | |
| --- | --- | --- | --- |
| Identity of the respondent | Tea set product sales staff 2 | Gender | male |
| Years of relevant work experience | 16 years | Education Qualifications | graduate degree |
| Place of interview | Offline: face to face | | |
| **Summary and record of interview key points** | | | |
| In my daily sales work, I often receive some comments and feedback from consumers about tea sets. These comments are both positive and negative. Whether the tea set product is easy to use, whether it is resistant to wear and fading, whether it is not easily outdated, and many other factors will affect consumers' satisfaction after purchase. Some customers complain that certain tea sets are beautiful in appearance but not practical. When using them, they always fall over with a slight touch, leaving tea all over the tea table. There are also some tea sets that do not have handles in order to look delicate, which will make your hands hot when you pick them up to drink tea. The material of the tea set failed to insulate the heat. Some customers also reported that the handles of some teapots are not convenient for pouring tea, and their hands have to be tilted at a large angle to pour tea. Usually, innovative tea sets with unique shapes placed on the display shelves in the store are most likely to attract consumers. Young customers prefer to buy personalized tea sets. Therefore, customers are paying more and more attention to the youthful, fashionable, interesting, and personalized appearance of tea set products. Regarding colors, many people prefer sober colors because they say they are easier to match with the decoration style of their homes. In recent years, customers have indeed begun to be keen on tea set products with Chinese cultural characteristics. For example, some tea set products that are designed to be related to traditional Chinese wedding culture or historical relics will have better sales and reviews. | | | |
